# Prognostic importance of survivin in breast cancer

**DOI:** 10.1038/sj.bjc.6600776

**Published:** 2003-04-01

**Authors:** S M Kennedy, L O'Driscoll, R Purcell, N Fitz-simons, E W McDermott, A D Hill, N J O'Higgins, M Parkinson, R Linehan, M Clynes

**Affiliations:** 1 Department of Pathology and Research Foundation, Royal Eye and Ear Hospital, Adelaide Road, Dublin 2, Ireland; 2 Department of Pathology, St Vincent's University Hospital, Elm Park, Dublin 4, Ireland; 3 National Institute for Cellular Biotechnology, Dublin City University, Glasnevin, Dublin 9, Ireland; 4 Department of Surgery, University College Dublin, Dublin, Ireland; 5 St Vincent's University Hospital, Elm Park, Dublin 4, Ireland

**Keywords:** apoptosis, breast cancer, IAPs, immunohistochemistry, prognostic indicator, survivin

## Abstract

Survivin is a member of the inhibitor of apoptosis (IAP) family, and is also involved in the regulation of cell division. Survivin is widely expressed in foetal tissues and in human cancers, but generally not in normal adult tissue. This study examined the expression of surviving protein in a series of 293 cases of invasive primary breast carcinoma. Survivin immunoreactivity was assessed using two different polyclonal antibodies, and evaluated semiquantitatively according to the percentage of cells demonstrating distinct nuclear and/or diffuse cytoplasmic staining. Overall, 60% of tumours were positive for survivin: 31% demonstrated nuclear staining only, 13% cytoplasmic only, and 16% of tumour cells demonstrated both nuclear and cytoplasmic staining. Statistical analysis revealed that survivin expression was independent of patient's age, tumour size, histological grade, nodal status, and oestrogen receptor status. In multivariate analysis, nuclear survivin expression was a significant independent prognostic indicator of favourable outcome both in relapse-free and overall survival (*P*<0.001 and *P*=0.01, respectively). In conclusion, our results show that survivin is frequently overexpressed in primary breast cancer. Nuclear expression is most common and is an independent prognostic indicator of good prognosis.

Apoptosis is the process whereby senescent or damaged cells are eliminated. It is a multistep cascade regulated by proteins that promote or counteract cell death. It is believed to be an important mechanism by which therapeutic chemotherapy and radiation therapy kill cancer cells. Aberrant inhibition of apoptosis interferes with normal cell regulation and promotes tumour development (for review, see Reed, 2001).

Survivin regulates cell division and inhibits apoptosis (Ambrosini *et al*, 1998). It is a member of the inhibitor of apoptosis (IAP) family, which have been shown to inhibit activated caspases, the cell death proteases either by acting directly (LaCasse *et al*, 1998; Deveraux and Reed, 1999; Shin *et al*, 2001) or indirectly (O'Connor *et al*, 2000; Reed, 2001).

Survivin mRNA was found to be diffusely expressed during foetal development, but unusually, among the IAP family, it was generally not found in normal adult tissues. In the majority of cancers studied to date, survivin is associated with poor prognosis. Survivin is overexpressed in most human cancers including bladder (Swana *et al*, 1999), blood (Adida *et al*, 2000a,2000b), colon (Kawasaki *et al*, 1998; Sarela *et al*, 2000), liver (Ito *et al*, 2000), brain (Nakagawara, 1998; Islam *et al*, 2000a), lung (Monzo *et al*, 1999), pancreas (Satoh *et al*, 2001), prostate (Xing *et al*, 2001), and kidney (Takamizawa *et al*, 2001). Although most immunohistochemical studies show survivin predominantly located in the cytoplasm, in some tumours survivin may have a mainly nuclear cellular location by immunohistochemistry (Ito *et al*, 2000; Okada *et al*, 2001), and its expression in the nucleus may be associated with a more favourable outcome (Okada *et al*, 2001).

Clinicopathological investigations on the role of survivin in breast cancer focusing on its importance as a prognostic factor have been limited. In this study, we investigated the prevalence and cellular localisation of survivin in a consecutive retrospective series of 293 primary breast cancers.

## MATERIALS AND METHODS

### Patient selection

The study material was derived from 293 consecutive cases of primary breast cancer, on which clinical follow-up and pathologic material, including snap-frozen tissue, were available for analysis from the 1993–1997 files of St Vincent's University Hospital Pathology Department, Dublin, Ireland. The patients involved underwent potentially curative resection at the hospital. A number of clinical and pathologic parameters were abstracted from patients' charts including details on age, postoperative treatment and follow–up, tumour stage, and hormonal analysis. Pathologic material was examined on each case by SK. Tumours were typed as described by Page and Anderson (1987) and graded as described by Elston and Ellis (1991). Staging was performed according to the TNM system of the UICC (Sobin, 1997).

### Immunohistochemistry

Formalin-fixed, paraffin-embedded tissue sections were immunostained for survivin. Sections 4 *μ*m thick were dewaxed in xylene, rehydrated in alcohol, and blocked for endogenous activity (3% H_2_O_2_ and normal rabbit serum). Antigen retrieval was carried out by pressure cooking in citrate buffer, pH 6.0. The sections were then incubated overnight at 4°C with polyclonal antibody AB469 (Novus Biologicals, Littleton, CO, USA) at dilution of 1 : 400 for all cases. Eight tumours were also investigated using the polyclonal antibody Surv 11A (Alpha Diagnostics, San Antonio, Texas, USA) at a dilution of 1 : 25 in order to check that the staining pattern was the same using a different antibody. Sections were washed in PBS pH 7.4 to remove unbound antisera. Bound antibody was detected using ABC detection kit (Vector Laboratories, Burlingame, CA, USA) with DAB as a chromogen. Slides were then lightly counterstained with Crazzi's haematoxylin.

Immunohistochemistry was also performed on 11 tumours snap frozen at −60°C. Sections 5 *μ*m thick were cut and air-dried for 2 h, fixed in acetone for 10 min, transferred to PBS, pH 7.4, and blocked with normal serum. Following optimisation of the antibody for frozen tissue, the sections were incubated with 1 : 150 dilution of primary antibody (Novus Biologicals) overnight at 4°C. Human melanoma sections were used as positive controls and included in each batch of 20 slides. An additional positive control used was a breast carcinoma shown to contain survivin mRNA by RT–PCR analysis. As a negative control, duplicate sections were stained without exposure to primary antibodies.

### Evaluation of immunohistochemistry results

Survivin immunoreactivity was evaluated semiquantitatively according to the percentage of cells demonstrating distinct nuclear and/or diffuse cytoplasmic immunohistochemical reaction. Nuclear and cytoplasmic tumour cell immunoreactivities were separately assessed in at least five high-power fields at × 40 magnification and assigned an arbitrary score as follows 0<5%; 1=5–20%; 2=21–50%; 3=51–75%; 4>76%. A cutoff value of >20% was established as a positive result. Invasive tumours with a score of 0 or 1 were considered negative. The results were separately analysed and statistically analysed. Two observers (SK, RP) separately scored the cases and agreed upon any discrepancies at a double-headed microscope.

### Statistical analysis

Descriptive statistics were used to summarise patient characteristics and statistical analysis of the results was performed using Pearson's *χ*^2^ test to demonstrate the relation between immunohistochemical and histopathologic findings. Kaplan–Meier survival curves were established and subsequently checked by log-rank, Breslow and Tarone-ware tests (*P*-values represent log-rank, unless otherwise indicated) to assess the prognostic significance of survivin expression in tumour cells. A value of *P*<0.05 was considered statistically significant. Multivariate analysis was performed with Cox regression model to assess additional prognostic values of the different variables. Statistical analyses were performed using Stata software (http://www.stata.com/).

## RESULTS

### Patient characteristics

The patients were aged between 31 and 90 years at the time of diagnosis (mean=57.7 years). Ninety-two women were less than 50 years and 201 women were over 50 years at diagnosis. The size of the tumours varied between 0.6 and 9 cm (mean=2.67 cm). Fifty-one tumours were T1 (<2 cm) in maximal dimension; 223 tumours were T2 (2–5 cm) and 19 tumours were T3 (>5 cm). Two hundered and forty-two tumours were invasive ductal carcinoma, NOS; 39 were invasive lobular; and 12 were tumours of special type (two tubular and 10 mucinous).

Twenty-four tumours were grade 1, 130 were grade 2, and 139 were grade 3. One hundred and ninety-four tumours were oestrogen receptor positive and 87 were oestrogen receptor negative (Oestrogen receptor status was determined by enzyme immuno-assay (EIA). A positive result was defined as more than 200 fmol(g protein(^−1^).). Oestrogen receptor status was not available for 12 patients. One hundred and thirty tumours had no axillary metastases and 163 tumours had metastasised to axillary lymph nodes.

One hundred and sixty-four women were treated with postoperative tamoxifen, 111 did not receive tamoxifen. One hundred and forty-six patients were treated with adjuvant systemic chemotherapy (CMF +/− adriamycin). One hundred and thirty-two patients did not receive chemotherapy. Details regarding tamoxifen and systemic chemotherapy were not available for 18 and 15 patients, respectively. Maximal follow-up was 3471 days with a mean follow-up of 1997 days (66.5 months). Six women were lost to follow-up less than 1 year postsurgery. One hundred and twenty-five patients had documented systemic metastases, 95 of whom died of disease and six patients had local recurrence. The details are summarised in Table 1

**Table 1:** Characteristics of 293 patients and univariate significance for relapse-free survival of selected parameters

**Characteristic**	**Number of patients**	**%**	**P-valu**e^a^
*Age* (years)			
⩽60	172	59	n.s.
>60	121	41	
			
*Histological grade*			
I+II	154	53	<0.0001
III	139	47	
			
*Lymph node status*			
Node −	130	44	0.0001
Node +	163	56	
			
*ER status*			
+(⩾10 fm mg^−1^)	194	66	0.0001
−(<10 fm mg^−1^)	87	30	
Unknown	12	4	
			
*Tumour size*^b^ (cm)			
⩽2.8	146	50	0.002
>2.8	147	50	
			
*Nuclear survivin* (%)			
>20	139	47	0.0033
⩽20	154	53	
			
*Cytoplasmic survivin*			
Pos	85	29	n.s.
Neg	208	71	
			
*Adjuvant chemotherapy*			
Yes	146	50	n.s.
No	132	45	
Unknown	15	5	
			
*Tamoxifen*			
Yes	164	56	n.s.
No	111	38	
Unknown	18	6	

a Log-rank test.

b 2.8 cm=median tumour size. n.s.=not significant.

.

### Immunohistochemical analysis

Specific staining for survivin was observed in tumour cells in 176 (60%) tumours. One hundred and seventeen tumours (40%) did not express survivin above the cutoff value of 20%. The breakdown of the distribution of survivin in tumours was as follows: 38.7% score 1; 26.3% score 2; 25% score 3; and 10% score 4.

**Figure 1:**
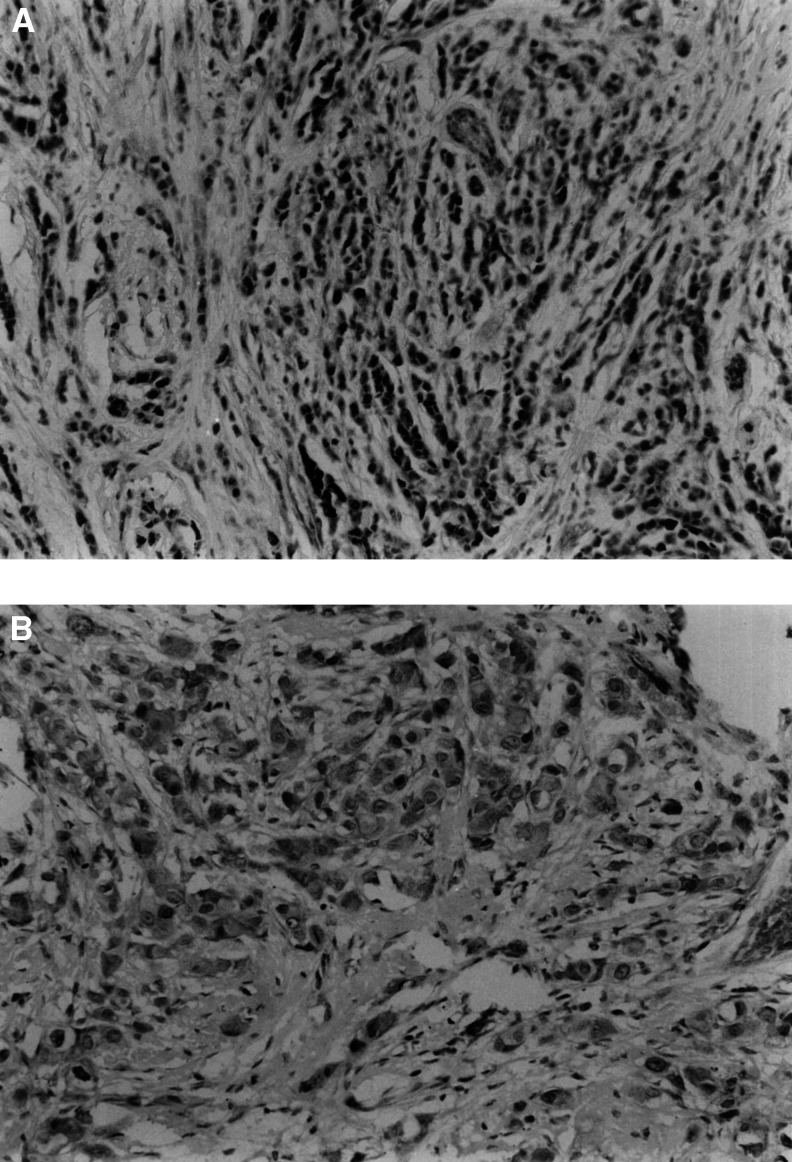
Infiltrating tumour cells showing (**A**) distinct nuclear positivity with polyclonal antibody to survivin (× 200 magnification) and (**B**) both nuclear and cytoplasmic positivity with antisurvivin antibody (× 400 magnification).

We found that of the specimens that were survivin positive, 139 of 176 expressed survivin in the nuclear region of the tumour cell. Figure 1A

illustrates a typical example. In 91 specimens, the reactivity was confined to the nucleus, and in 48 it was present in both nucleus and cytoplasm as seen in Figure 1B. In 37 positive cases, survivin was expressed only in the cytoplasm. Nuclear and cytoplasmic expression was occasionally seen in ductal carcinoma *in situ*. There was focal, weak expression in some normal ducts and lobules in cases where tumour cells showed strong immunoreactivity. Stromal cells did not express survivin, but peritumoral lymphocytes did react with survivin antibody in some cases.

The immunohistochemical study of 11 frozen sections of cases revealed unequivocal positivity in 10 cases. Seven cases had immunoreactivity of tumour cells in the nucleus alone. In three cases, there was nuclear and cytoplasmic positivity in tumour cells. In each case, the pattern correlated with that of the corresponding formalin-fixed, paraffin-embedded tissue.

Eight formalin-fixed paraffin-embedded tumour cases also were examined with a second survivin antibody (Alpha Diagnostics). In three cases, immunoreactivity was confined to the nucleus; in three cases, it was confined to the cytoplasm; and in two cases, there was both cytoplasmic and nuclear immunoreactivity. These results correlated with those of the Novus antibody in seven of the eight cases.

### Statistical analysis

To evaluate the prognostic significance of survivin expression at diagnosis, survivin expression was analysed in relation to relapse-free survival (RFS) and Overall survival. In a Cox univariate analysis for RFS in all patients, nuclear survivin <20% was significantly associated with a shortened RFS. Similarly, grade, ER status, nodal status, and size were significantly related to RFS in univariate analysis. On the other hand, cytoplasmic survivin expression, age at diagnosis, and treatment (adjuvant or hormonal therapy) were not significantly related to RFS in univariate analysis (Table 1). There was no significant association between nuclear survivin expression levels and any other clinicopathologic feature analysed (Table 2

**Table 2:** Correlation between clinicopathologic factors and expression of nuclear survivin in breast cancer

	**Nuclear survivin expression**		
	**Negative**^a^ (*n*=154)	**Positive**^b^ (*n*=139)	***P*-value**
*Age* (years)			
⩽60	93	89	n.s.
>60	61	50	
			
*Histological grade*			
I+II	75	79	n.s.
III	79	60	
			
*Lymph node status*			
Node −	76	88	n.s.
Node +	78	51	
			
*ER status*			
+ (⩾10 fm mg^−1^)	96	99	n.s.
− (<10 fm mg^−1^)	51	35	
Unknown	7	5	
			
*Tumour size* (cm)			
⩽2.8	77	71	n.s.
>2.8	77	68	
			
*Adjuvant chemotherapy*			
Yes	76	70	n.s.
No	68	64	
Unknown	10	5	
			
*Tamoxifen*			
Yes	91	73	n.s.
No	51	60	
Unknown	12	6	

a ⩽20% nuclear survivin expression.

b >20% nuclear survivin expression. n.s.=not significant.

). There was no statistical significance when looking at the effect of chemotherapy on survival between survivin-negative and survivin-positive tumours.

In multivariate analysis, the most important prognostic factors for disease-free survival were negative lymph node status (*P*<0.001), size (*P*=0.046), nuclear survivin expression (cutoff point=20% (*P*<0.001)), and oestrogen receptor status among lymph node-positive patients (*P*=0.042) (Table 3

**Table 3:** Multivariate analysis for disease-free survival

**Variables**	**Hazard ratio**	**s.e.**	**Coefficient**	***P*-value**
Nodal status	3.295789	1.069509	3.68	<0.001
ER^a^	0.7868536	0.2716365	−0.69	0.487
ER × LN positive^b^	0.4310035	0.1781777	−2.04	0.042
Size^c^	1.477919	0.289276	2.00	0.046
Grade^d^	1.466601	0.2992795	1.88	0.061
Nuclear survivin^e^	0.4846783	0.094241	−3.72	<0.001

a Estrogen receptor positive *vs* negative.

b Effect of ER status on survival where lymph node metastases have occurred.

c Dichotomised size variable using the median (2.8 cm) as the cutoff.

d Grades I and II grouped together vs grade III.

e Dichotomised nuclear survivin, cutoff=20%.

). The Kaplan–Meier survival curves demonstrate the better prognosis of nuclear survivin-positive tumours for disease-free (Figure 2A

**Figure 2:**
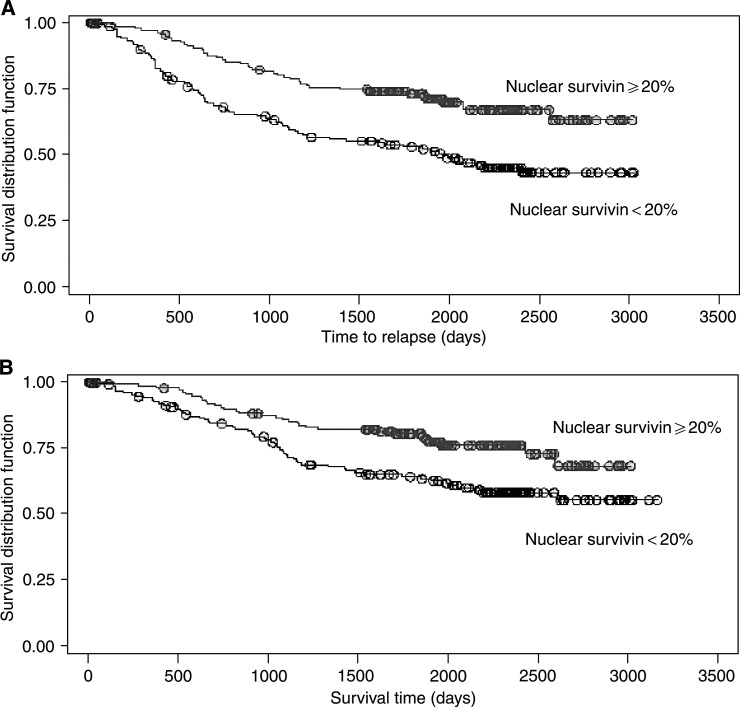
Kaplan–Meier estimates for (**A**) disease-free survival and (**B**) overall survival, by nuclear survivin <20 *vs* ⩾20%, adjusted for cytoplasmic result, oestrogen receptor status, lymph node status, grade, and size.

) and overall survival (Figure 2B).

In terms of overall survival, the most important prognostic factors were size (*P*=0.016), grade (*P*=0.012), nuclear survivin expression (*P*=0.010), and lymph node status (*P*=0.044) (Table 4

**Table 4:** Multivariate analysis for overall survival

**Variables**	**Hazard ratio**	**s.e.**	**Coefficient**	***P*-value**
Nodal status	1.625208	0.3916559	2.02	0.044
Size^a^	2.335183	0.8202764	2.41	0.016
Grade^b^	1.790949	0.4176715	2.50	0.012
Nuclear survivin^c^	0.5663471	0.1249786	−2.58	0.010

Model is stratified using quartiles of age.

a Dichotomised size variable using the median (2.8 cm) as the cutoff.

b Grades I and II grouped together *vs* grade III.

c Dichotomised nuclear survivin, cutoff=20%.

). The hazard ratios in the multivariate Cox models give the risk of relapse/death adjusted for time and the other covariates, for example, lymph node status. This analysis shows that patients whose tumours have nuclear survivin in >20% of tumour cells have approximately half (0.5663) the risk of death or relapse compared to the women whose tumours have negative or below cutoff level of nuclear survivin.

Nuclear survivin expression is an independent favourable prognostic indicator for both disease-free and overall survival. The effect of cytoplasmic positivity alone shows a similar trend, but is less significant as numbers of positive cases are smaller. The relative risk of death or systemic relapse in patients whose tumours are survivin negative compared with those whose tumours are survivin positive is 1.76.

## DISCUSSION

The aim of our study was to investigate the expression of survivin protein in invasive breast carcinoma by immunohistochemistry.

Survivin protein was detected in 60% of tumours. It was most commonly located in the nucleus (139 of 176 positive cases), but was seen in a minority (85 of 176 positive cases) of tumours in the cytoplasm. In multivariate analysis, the presence of survivin protein in invasive breast cancers is a strong independent prognostic indicator of 5-year RFS and overall survival.

A lack of consensus exists regarding the importance of intracellular location and prognostic significance of survivin expression in common epithelial tumours. Survivin was originally reported by Ambrosini *et al* (1997) to be present during foetal life but undetectable in adult differentiated tissues (Ambrosini *et al*, 1997). It is also expressed at mRNA and protein level in a range of common epithelial tumours including colon (Sarela *et al*, 2000), pancreas (Satoh *et al*, 2001), prostate (Xing *et al*, 2001), and breast (Tanaka *et al*, 2000). As a result of this widespread expression in tumours, and generally low-level expression in normal tissue, survivin was considered to be a potentially valuable new target for apoptosis-based chemotherapy. Subsequent studies have shown that survivin is expressed in the normal endometrium (Konno *et al*, 2000). It is expressed in the nucleus and cytoplasm of basal and glandular cells, in a pattern correlating with proliferative and secretory phase of the menstrual cycle. Survivin expression has been found in normal colon as well as in hyperplastic polyps, adenomatous polyps, and colonic adenocarcinoma (Gianani *et al*, 2001).

Our findings that survivin subcellular location may be predominantly nuclear in breast carcinoma and that its presence may be a favourable prognostic indicator are at variance with one previous report of the significance of survivin protein expression in invasive breast carcinoma. The study by Tanaka *et al* (2000) used an antibody (mAb 8E2, provided by Dr DC Altieri, Yale University, CT, USA) different from the one we used. There is a suggestion that this antibody may detect cytoplasmic survivin only (Fortugno *et al*, 2002). Their results show a trend towards association between cytoplasmic survivin and a bad prognosis. This was not statistically significant. They found that apoptotic index (AI) is an independent prognostic indicator and they found a significant correlation between AI and cytoplasmic survivin. They inferred from this a relation between cytoplasmic survivin and poor prognosis. It is also possible that other apoptotic factors may have contributed to the decreased AI in the tumours that relapsed. Our results show a statistically significant relation between nuclear survivin expression and good prognosis.

Our study showed no correlation between survivin expression and other clinicopathological prognostic features including tumour size. Our study population only included 17% pT1 tumours, but there were 95 (35%) tumours ⩽2 cm and a linear relation exists between size increase and probability of relapse. In our study, we found that there was strong evidence of a correlation between high levels of nuclear survivin and increased survival time to relapse or death.

Okada *et al* (2001) reported survivin expression in the nucleus as well as in the cytoplasm in gastric cancer. Nuclear localisation was associated with favourable prognosis in multivariate analysis. Cytoplasmic survivin expression was associated with a poor prognosis. In a study of hepatocellular carcinomas, survivin expression was mainly in the nucleus with weak cytoplasmic staining of tumour cells and no expression in normal tissues (Ito *et al*, 2000). Also, survivin did not significantly correlate with the AI as calculated by the TUNEL method in hepatocellular carcinoma.

Survivin has been described both as a chromosomal passenger protein in the nucleus and as a cytoplasmic microtubule-associated protein. Survivin is normally expressed in the G2/M phase of the cell cycle in a cell-cycle-regulated manner, and is associated with microtubules of the mitotic spindle during mitosis (Li *et al*, 1998). Disruption of survivin–microtubule interactions has been proposed to result in loss of survivin's antiapoptosis function and increased caspase-3 activity during mitosis (Tamm *et al*, 1998; Li *et al*, 1999).

Fortugno *et al* (2002), in a study using a novel panel of monoclonal and polyclonal antibodies, have shown that there are different subcellular pools of survivin. A nuclear pool that segregates with nucleoplasmic proteins was identified. A separate and predominant cytosolic pool associates with interphase microtubules, centrosomes, spindle poles, and mitotic spindle microtubules at metaphase and anaphase. The two survivins are immunochemically distinct, independently modulated during cell cycle progression and only cytosolic survivin associates with p34^cdc2^. Phosphorylation of survivin by p34^cdc2^–cyclin B has been identified as a requisite for apoptosis inhibition (O'Connor *et al*, 2000). The postulated explanation for these findings was that separate post-translational modifications could differentially affect epitope accessibility of nuclear *vs* cytosolic microtubule-bound survivin *in vivo*. If nuclear survivin cannot associate with p34, an essential step in apoptosis inhibition, it may actually induce apoptosis. This may explain why different patterns of survivin localisation are seen in different tumour types and may partly explain the different prognostic implications of cytoplasmic and nuclear survivin. Survivin may be only effective in blocking apoptosis when located in the cytosol where caspases are predominantly located. Nuclear survivin must be phosphorylated for binding to processed caspase-9. A nonphosphorylatable alanine (T34A) mutant of survivin has been described, which disrupts cell division and induces apoptosis, probably by substrate competition (O'Connor *et al*, 2000).

Recently, splice variants of survivin with different antiapoptotic properties have been identified (Mahotka *et al*, 1999; Fogt *et al*, 2001; Krieg *et al*, 2002). One of these variants, survivin-2B, has reduced antiapoptotic potential and may act as a naturally occurring antagonist of survivin. In a study of gastric carcinomas, all gastric carcinomas were found to express mRNA encoding the splice variants survivin delta EX 3 and survivin-2B as well as survivin. In that study, there was a decrease in survivin-2B mRNA in higher stage disease. Polyclonal antibodies, such as the antibodies used in the current study, will react with all survivin variants except the delta EX 3 variant with the Alpha Diagnostics antibody. Our results showed a good correlation between two antibodies on a limited panel of formalin-fixed and fresh breast cancer tissue.

Our data show that polyclonal antibodies to survivin detect both cytoplasmic and nuclear forms of survivin in breast cancer. The nuclear form is most common and is an independent prognostic indicator of good outcome. This is surprising in view of reports on other common epithelial tumour types showing that survivin expression is associated with a worse outcome, but has analogies with the other main apoptosis inhibitor bcl-2 whose overexpression is associated with a better outcome in breast cancer and a favourable response to therapy (Leek *et al*, 1994; Duenas-Gonzalez *et al*, 1997). The reason for this apparent contradiction lies in the interaction of bcl-2 with other family members, for example, Bax. The ratio of Bcl-2 to Bax and the ability of Bcl-2 to form homo- and heterodimers holds the key to the regulation of the balance between cell survival and apoptosis. We hypothesise that survivin is of a similar nature, that different splice variants may interact, and subcellular location ratio of the protein may contribute to a complex regulation of apoptosis. This hypothesis is supported by the suggestion that survivin-2B may impair or oppose the function of survivin, acting in a dominant-negative manner (Islam *et al*, 2000b). Our results are in agreement with the reported study of gastric carcinomas (Okada *et al*, 2001), where nuclear staining is associated with favourable prognosis.

Intracellular location of survivin may have an important physiologic role in the cell cycle and have different prognostic implications, as in the case of cyclin D2. Nuclear localisation of cyclin D2 has been reported to have good prognostic import. It is associated with well-differentiated tumours, lower depth of cancer invasion, fewer lymph node metastases, and less vessel invasion. In contrast, cytoplasmic location of cyclin D2 is associated with a poor prognosis (Takano *et al*, 1999,^2000^).

Absence of survivin in node-negative breast cancer patients may herald a higher risk of relapse and a shorter survival. Further studies on breast cancer, when selective antibodies become available, may elucidate the role of survivin, including its location and possibly antagonistic roles of splice variants in apoptosis inhibition and cell cycle control in breast cancer.
